# Combined Effects of Gaze and Orientation of Faces on Person Judgments in Social Situations

**DOI:** 10.3389/fpsyg.2017.00259

**Published:** 2017-02-22

**Authors:** Raphaela E. Kaisler, Helmut Leder

**Affiliations:** ^1^Cognitive Science Research Platform, University of ViennaVienna, Austria; ^2^Department of Basic Psychological Research and Research Methods, University of ViennaVienna, Austria

**Keywords:** three-quarter views of faces, gaze direction, person judgments, attractiveness, trustworthiness, social scene

## Abstract

In social situations, faces of others can vary simultaneously in gaze and orientation. How these variations affect different kinds of social judgments, such as attractiveness or trustworthiness, is only partly understood. Therefore, we studied how different gaze directions, head angles, but also levels of facial attractiveness affect perceived attractiveness and trustworthiness. We always presented pairs of faces – either two average attractive faces or a highly attractive together with a less attractive face. We also varied gaze and head angles showing faces in three different orientations, front, three-quarter and profile view. In Experiment 1 (*N* = 62), participants rated averted gaze in three-quarter views as more attractive than in front and profile views, and evaluated faces with direct gaze in front views as most trustworthy. Moreover, faces that were being looked at by another face were seen as more attractive. Independent of the head orientation or gaze direction, highly attractive faces were rated as more attractive and more trustworthy. In Experiment 2 (*N* = 54), we found that the three-quarter advantage vanished when the second face was blurred during judgments, which demonstrates the importance of the presence of another person-as in a triadic social situation-as well as the importance of their visible gaze. The findings emphasize that social evaluations such as trustworthiness are unaffected by the esthetic advantage of three-quarter views of two average attractive faces, and that the effect of a faces’ attractiveness is more powerful than the more subtle effects of gaze and orientations.

## Introduction

In social interactions, the perceivers can make various evaluations, such as judging attractiveness or trustworthiness. In natural social situations several variables vary simultaneously such as gaze directions or head orientations, and these might affect the evaluations of others differently. Such interactions are interesting, because they reveal the complexity of social interactions by showing how person evaluations are based on different underlying processes. Therefore, studies need to consider varying cues simultaneously to investigate combined effects in social interaction.

In the present study we were particularly interested if face processing is modulated by interactions of rather social cues, such as gaze direction (Baron-Cohen, 1995; Emery, 2000), and the orientation of faces, especially the often-considered attractive three-quarter view (Bruce et al., 1987), when judging the attractiveness and trustworthiness of others. In order to study social perception we employed a design in which a perceiver saw pictures of realistic real world scenes always showing two other people, a situation as in an everyday life triadic interaction. In particular, we studied three variables that play a role when perceiving two people in a social scene. First, the direction of gaze, since it has been previously shown that people often prefer direct gaze and consider it as attractive (Ewing et al., 2010). Moreover, being looked at by another person is preferred and affects as how attractive or trustworthy this person is evaluated (Kaisler and Leder, 2016). Second, we systematically varied head orientations: when people interact with each other, they automatically turn their head in order to look at and engage with others. Head orientation is also known to affect person evaluations: in particular, the three-quarter view of a face is often considered as attractive (Bruce et al., 1987). And third, we varied facial attractiveness as a feature of a person, which captures attention and elicits positive emotions (Hayden et al., 2007) that might lead to engagement or disengagement with others. Thus, in social situations, we simultaneously perceive systematic variations of gaze direction and head orientations that might interact with a person’s facial attractiveness in our evaluation of others.

### Gaze Effects

Direct gaze positively affects the judgment of attractiveness (Macrae et al., 2002; Mason et al., 2005; Ewing et al., 2010) and trustworthiness (Bayliss and Tipper, 2006; Todorov et al., 2013) People consider direct gaze as attractive and trustworthy and prefer direct gaze over averted gaze. Gaze further affects the liking of objects, as Bayliss et al. (2006) showed with increased liking of objects that are gazed at by a face. Based on these findings, Kaisler and Leder (2016) replicated such higher liking with faces in triadic social scenes. Participants judged faces that were looked at by a second face as more trustworthy than when they were not gazed at.

### Facial Attractiveness

Attractive faces affect social evaluations of others in various ways. Attractiveness plays a primary role in impression formation and captures attention (Lindell and Lindell, 2014). It biases our perception of direct eye contact (Kloth et al., 2011), and induces a pleasurable perceptual experience. Attractiveness alters our behavior, so that we look longer at attractive faces (Aharon et al., 2001; Levy et al., 2008) in social scenes (Leder et al., 2010; Mitrovic et al., 2016). Attractive faces may be looked at longer because looking at them is rewarding and they elicit positive emotions (Hayden et al., 2007). In order to understand the effect of different viewing angles on face evaluation and its dependence on facial attractiveness, we also included different levels of facial attractiveness-highly, average and less attractive faces. This allowed us to test whether and to what amount the attractiveness of a face is modified by variations in viewing angle and gaze direction. Attractiveness is often positively related with other social evaluations for example, judging whether a person is trustworthy results from automatic rapid processing of facial attributions (Todorov et al., 2015). In the present study, we investigated gaze and head angle effects in dependence of different levels of facial attractiveness, and how these variables interact and affect our social evaluations.

### Head Angles

Different head angles and viewpoints change our perception and evaluation of others. The orientation of faces influences our perceived direction of gaze (Langton and Bruce, 2000; Langton et al., 2000). Rule et al. (2009) investigated attractiveness and trustworthiness judgments from three-quarter and profile views, as compared to front views. They found a drop in correlation of profile with front and three-quarter view judgments of trustworthiness, but not attractiveness, and consistency of ratings among viewing angles given unrestricted viewing-time. In front views (0°), internal features of the face-such as the eyes, nose, and mouth-are fully visible. In three-quarter views (45°), similar features with even more information are available. For example, in three-quarter views, the protuberance of the nose and most facial features can be seen, and this results in better recognition of unfamiliar faces than with profile or front views (Bruce et al., 1987; Hill and Bruce, 1996; McKone, 2008). However, profile views (90°)-which may result when someone looks at someone else-limit recognition of important characteristics, e.g., pertaining to the eyes, because certain information cannot be assessed, such as the interocular distance, and the eyebrow-shape/size (Leder and Bruce, 2000). This may result, among other factors, in a decline in recognition accuracy of profiles relative to three-quarter and front views of unfamiliar faces and therefore may reduce the effectiveness of gaze cues relative to other views (Stephan and Caine, 2007). Other features may also influence our perception of faces, such as the property of an image and the within-person variability among different images, as well as changes in expression, age, and health among others (Burton et al., 2015). In our study, we controlled for distance, angle, luminosity, facial expression, age, and model’s clothing; we kept natural variation to a minimum but systematically varied head angles, gaze directions, and facial attractiveness. We investigated how different viewing angles, gaze directions and the interaction of these two variables affect the perceived attractiveness and trustworthiness (Experiment 1).

### Interactions

In this study, we are particularly interested in the interaction of effects, as people perceive gaze, head orientation and facial attractiveness simultaneously. Each of these variables influences our social evaluation to some extent, but the presence of another person-a second person in a scene-then additionally contributes to our judgment formation. The presence of the second person might also enhance combined effects of gaze and orientation. For example, people in groups are considered more attractive than in isolation (Walker and Vul, 2014; Kaisler and Leder, 2016). Moreover, when the observer looks at a scene containing two individuals, these two individuals also show a certain kind of interaction: looked at by others (Kaisler and Leder, 2016). Therefore, in the present study, we systematically investigate combined effects of gaze and orientation in triadic social situations. Our experimental design included the following factors: the observer (the participant), and two faces in a perceived scene, one face that is looking and one face that is being looked at. We systematically varied gaze and head angles accordingly with highly, average and less attractive faces to investigate their influence on observer’s judgments. For highly attractive faces we naturally expected high judgments of attractiveness, but also for trustworthiness, because of attentional and reward related advantage (Hayden et al., 2007).

The variation of viewing angles allowed us to test for effects of head orientation and its interaction with gaze effects; for example, direct gaze in front view is considered as more attractive in dyadic (e.g., Ewing et al., 2010) and triadic social scenes (Kaisler and Leder, 2016). We expected higher attractiveness judgments for three-quarter views, due to their procedural advantage, when accompanied with direct gaze in front view, thereby eliminating the direct gaze bias. This emphasizes that the presence of a second person, and therefore the comparison of facial attractiveness to this second person would further enhance this orientation effect. We expected no relationship between the viewing angle and gaze direction for trustworthiness judgments, resulting in a direct gaze bias. Regarding profile views, we expected a preference for direct gaze (0°) in both judgments, due to their limited information availability regarding the eyes.

As people make social judgments very quickly (Willis and Todorov, 2006), we tested whether judgments of facial attractiveness and perceived trustworthiness are differentially affected by observers in social interactions. The difference of these measures becomes clear when considering that both are related to a certain social context: judgments of attractiveness to mating related purposes, and judgments of trustworthiness to others’ explorative and communicative behavior in the situation. We measured facial attractiveness as an esthetic judgment, which to a large extent depends on individual preferences and is associated with social approach and mating (Leder et al., 2016). Face attractiveness furthermore relies on physiognomic features such as symmetry of the face and averageness (Langlois and Roggman, 1990; Perrett et al., 1999). We expected the perceived attractiveness to be sensitive to the other variables involved. Following this, increased attractiveness ratings can be hypothesized to occur for three-quarter views of faces, for highly attractive faces, and for averted gaze faces that were looked at by the second face, referred to as the “being looked at” gaze effect (Kaisler and Leder, 2016). However, this might not be relevant to social evaluations. We therefore also measured trustworthiness as a social evaluation, which due to a weaker relation with facial features (Todorov et al., 2008) might be particularly sensitive to social situations, i.e., the presence and looking behavior of others. Perceived trustworthiness isn’t based on distinct facial features (Todorov, 2008; Todorov et al., 2008), but seems to relate more to the typicality of a face (Oosterhof and Todorov, 2009; Sofer et al., 2015). However, people judge typical-i.e., average-faces as more attractive (Todorov et al., 2013).

**FIGURE 1:**
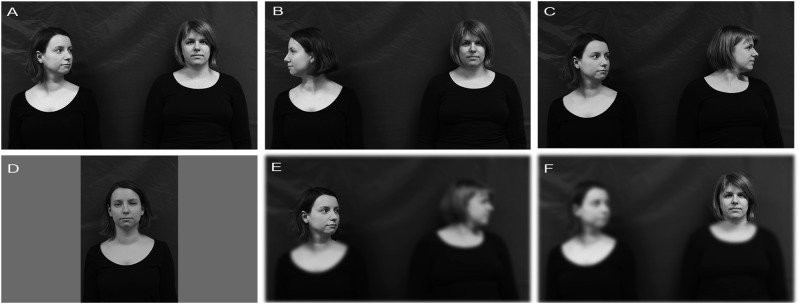
**Gaze conditions across orientation of faces. (A)** A social approach (DIR+LAT), an averted gaze face in three-quarter view (45°) looking *toward* a direct gaze in front view (0°), **(B)** a social avoidance condition (DIR+AV), an averted gaze face in profile view (90°) looking *away* from a direct gaze in front view (0°), and **(C)** an ambiguous gaze condition (LAT+AV), averted gaze face in three-quarter view (45°) looking *toward* while the second face looks *away* in profile view (90°). **(D)** One-face scene, the face shows direct gaze in front view toward the observer (in Experiment 1), and an example of stimuli in the rating block of Experiment 2; participants rate the left **(E)** and the right face **(F)** separately while the background is blurred.

In the present study, participants judged faces in pairs of either two average attractive, or one highly and one less attractive face, with varying gaze and head cues for attractiveness and trustworthiness. In two experiments we investigated the effects of facial attractiveness by varying the viewing angle and gaze direction independence of the individual attractiveness of a face (Experiment 1, **Figures 1A–D**). In Experiment 2, we investigated whether the three-quarter advantage vanishes when the second face has been blurred during judgments, stressing the importance of visible gaze, but also of the presence of another person (**Figures 1E,F**). We combined three gaze directions (direct, looking toward, looking away) with three head angles (0°, 45°, 90°) in two types of scenes: two average attractive faces, and one highly and one less attractive faces. **Figure 1** shows (A) a social approach (DIR+LAT), one face is looking *directly* (DIR, 0°) at the observer and the second face is *looking at* the second face (LAT, 45°), (B) a social avoidance (DIR+AV), the second face shows averted gaze *looking away* (AV, 90°) from the *direct* gaze face (DIR) and (C) an ambiguous situation (LAT+AV), both faces showing averted gaze from the observer’s point of view, one face is *looking at* (LAT, 45°) while the second face that in turn is *looking away* (AV, 90°). We chose the ambiguous gaze condition (LAT+AV) to be able to have stimuli without direct gaze toward the observer, and to test whether the gaze cue of the looking face (45°) enhances the preference for the looked-at face (90°).

## Experiment 1

### Materials and Methods

#### Participants

Sixty-two adult undergraduate psychology students from the University of Vienna participated in the experiment (25 of them male, *M*_age_ = 23.37, *SD* = 5.19). The experimenter explained the procedure to the participants and obtained written informed consent prior to the experiment in accordance with the ethical guidelines of the University of Vienna. Participants had normal or corrected-to-normal vision and received course credits for their participation in the experiment.

#### Stimuli

We produced 12 high-quality gray scale photographs of scenes containing pairs of female university students showing neutral face expressions. Scenes were pre-rated as expressing neutral emotional valence (Strick et al., 2008) and pre-selected so that in half of the scenes both models’ facial attractiveness differed less than 0.5 rating points (equal attractive scenes), and the other half differed more than 1.2 ratings points (different attractive scenes) on a seven point rating scale (separate pre-study). We photographed the models in front of a gray background, and placed two models side by side with a lateral separation of one meter from the midpoint of the torso in the scene (Kaisler and Leder, 2016). The models were photographed under conditions that controlled for distance, angle, and luminosity. In all gaze conditions, model’s gaze direction and head angle were matched, resulting in three gaze conditions: DIR+LAT, DIR+AV, and LAT+AV (**Figures 1A–C**). For isolated views, i.e., one-face-scenes, we took separate photographs of the models showing direct gaze in front view (0°) in the middle of a scene with gray background to obtain baseline measures (**Figure 1D**). The side (left–right) of the looking and looked at face was counterbalanced, resulting in a total of 96 stimuli (12 scenes with three gaze conditions and two left–right versions plus 24 one-face scenes).

#### Design and Procedure

Stimuli were presented on a 19″ LCD display at 1280 × 1024 pixel resolution and viewed from a distance of approximately 50–60 cm. Stimulus presentation, trial randomization, and data collection were conducted with E-Prime (V.2, Psychology Software Tools). Participants rated all 24 faces separately in two rating blocks (randomized for left- and right face and scene), once for attractiveness and once for trustworthiness. The order of the rating blocks was counterbalanced across participants and the stimuli randomized for trials and side. The instructions above the picture indicated whether the left or right face had to be rated. The original wording stated: “How attractive/trustworthy is the left/right person?” Participants were instructed to use a computer mouse to select a number on a seven point Likert scale presented below each scene, with “1” being “very unattractive” or “not at all trustworthy” and “7” being “very attractive” or “very trustworthy.” We instructed participants to respond as spontaneously as possible and encouraged them to use all the numbers available on the scale, as they deemed appropriate. The exposure duration of the picture was unlimited and the rating scale remained on screen until a response was given. Each participant thus rated each of the 24 faces presented in 12 scenes twice in a single session lasting approximately 40 min. After the rating blocks, participants filled in a questionnaire, which contained questions about the familiarity of the faces (data not shown). Because familiarity strongly affects the quality of face processing (see Burton et al., 2015), we excluded 10 participants who indicated that they knew any of the presented faces from data analyses.

### Results and Discussion

#### Analysis of Scene Types

In order to show the influence of facial attractiveness on person judgments of others, we analyzed gaze effects of equal and different attractive scenes. We calculated mean values of direct (DIR), looking (LAT), and averted gaze (AV) across three gaze conditions (**Table 1**). Reliability analyses indicated internal consistency of participant’s attractiveness ratings, Cronbach’s α = 0.96, and trustworthiness ratings, Cronbach’s α = 0.90 in scenes of equal attractiveness levels. Similar for scenes with different attractiveness levels, consistency between attractiveness ratings, Cronbach’s α = 0.93, and trustworthiness ratings, Cronbach’s α = 0.89. We conducted a 3x3x2 repeated measurement ANOVA (95% confidence interval) with gaze directions (DIR, LAT, AV) and scene type (equal attractive scenes: average; different attractive scenes: highly and less attractive faces) as within-subject factor and gender (male, female), as between-subject factors.

**Table 1:** Mean ratings of gaze conditions in Experiment 1.

Scene	Gaze direction	Attractiveness *M* (*SD*)	ANOVA		Trustworthiness *M* (*SD*)	ANOVA	
			*p*	$\eta_{p}^{2}$		*p*	$\eta_{p}^{2}$
**Equal attractive faces**							
DIR+LAT	Direct	3.60 (0.84)	0.001	0.52	**4.29 (0.77)**	0.005	0.12
	Looking at	**4.03 (0.78**)			4.05 (0.66)		
DIR+AV	Direct	**3.79 (0.91**)	0.001	0.34	**4.24 (0.73**)	0.001	0.37
	Averted away	3.41 (0.78)			3.63 (0.69)		
LAT+AV	Looking at	**3.80 (0.82)**	0.035	0.07	3.98 (0.71)	>0.05	–
	Averted away	3.65 (0.75)			3.96 (0.78)		
**Different attractive faces**							
DIR^∗^+LAT	Direct^∗^	**3.98 (0.96)**	0.001	0.69	**4.32 (0.91)**	0.001	0.47
	Looking at	2.69 (0.80)			3.37 (0.93)		
DIR^∗^+AV	Direct^∗^	**4.01 (0.92)**	0.001	0.63	**4.37 (0.83)**	0.001	0.39
	Averted away	3.03 (0.93)			3.55 (0.93)		
LAT^∗^+AV	Looking at^∗^	**4.84 (1.00)**	0.001	0.74	**4.48 (0.80)**	0.001	0.98
	Averted away	2.94 (1.04)			3.71 (0.90)		
DIR+LAT^∗^	Direct	2.83 (0.93)	0.001	0.69	4.00 (0.88)	0.001	0.17
	Looking at^∗^	**4.16 (0.99)**			**4.40 (0.73)**		
DIR+AV^∗^	Direct	2.86 (0.99)	0.001	0.68	3.95 (0.89)	>0.05	–
	Averted away^∗^	**4.45 (1.01)**			4.09 (0.86)		
LAT+AV^∗^	Looking at	2.85 (0.86)	0.001	0.55	3.45 (0.86)	0.001	0.24
	Averted away^∗^	**3.85 (0.92)**			**4.04 (0.96)**		

*It shows mean attractiveness and trustworthiness ratings of three gaze conditions in two scene types: two average attractive faces (equal attractiveness scene), and one highly and one less attractive face (different attractive faces). Bold face indicates significant differences revealed by ANOVA. Asterisk indicates highly attractive faces in scenes with different levels of facial attractiveness.*

^∗^*Highly attractive face; bold face, statistically significant (p < 0.05); $\eta_{p}^{2}$, effect size.*

**FIGURE 2:**
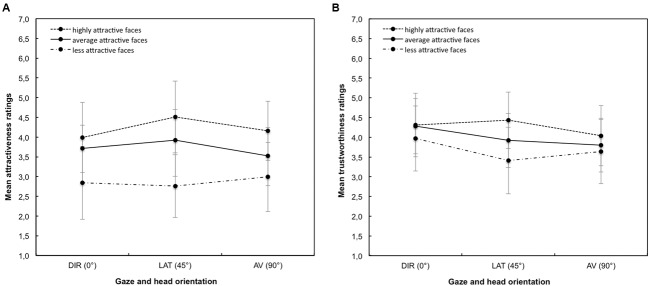
**Analyses of highly, average and less attractive faces in triadic situations. (A)** Shows mean attractiveness ratings of equal and different scenes. Participants rated highly attractive faces most attractive followed by average and less attractive faces. Note that averted faces looking *toward* (45°) are considered more attractive than direct (0°) and averted gaze looking *away* (90°). **(B)** Shows mean trustworthiness ratings of equal and different scenes. Participants rated highly attractive faces more trustworthy than average and less attractive faces. Note that direct gaze (0°) of average and less attractive faces are considered more trustworthy than averted gaze faces looking *toward* (45°) or *away* (90°) from a second face.

We found significant differences between the gaze direction and scene type for attractiveness judgments (**Figure 2A**): gaze, *F*(2,61) = 13.15, *MSE* = 2.23, *p* = 0.001, $\eta_{p}^{2}$ = 0.18, scene type, *F*(2,61) = 208.15, *MSE* = 84.17, *p* = 0.001, $\eta_{p}^{2}$ = 0.78, and an interaction between gaze and scene type, *F*(4,61) = 12.79, *MSE* = 2.09, *p* = 0.001, $\eta_{p}^{2}$ = 0.18. We found a similar pattern for trustworthiness judgments (**Figure 2B**): gaze, *F*(2,61) = 14.79, *MSE* = 5.46, *p* = 0.001, $\eta_{p}^{2}$ = 0.20, scene type, *F*(2,61) = 45.11, *MSE* = 15.58, *p* = 0.001, $\eta_{p}^{2}$ = 0.43, as well as an interaction between gaze and scene type, *F*(4,61) = 10.73, *MSE* = 2.06, *p* = 0.001, $\eta_{p}^{2}$ = 0.15. We did not find gender effects or interactions with gender. Therefore, further analyses were conducted separately for equal and different attractive scenes.

#### Analysis of Gaze Conditions

We analyzed participants’ judgments of three gaze conditions in which each face and scene were rated twice, for attractiveness and trustworthiness. For equal attractiveness scenes, a repeated measurement ANOVA (95% CI) of attractiveness judgments with gaze directions (left and right face) as within-subject factor, showed a preference for the looking faces (45°) in the social approach (DIR+LAT), for direct gaze faces (0°) in the social avoidance (DIR+AV), and for the looked-at face in the ambiguous gaze condition (LAT+AV, **Table 1**). However, regarding social evaluations, participants considered direct-gaze faces (0°) as more trustworthy in the social approach and avoidance condition. In scenes with different facial attractiveness levels, participants rated highly attractive faces as more attractive and trustworthy, independent of the gaze direction and head angle.

These findings emphasize the esthetic advantage of three-quarter views of faces when judging facial attractiveness (e.g., Langton and Bruce, 2000), hence participants considered faces looking toward (45°) the second face as being more attractive than direct-gaze (0°) faces. However, trustworthiness judgments were unaffected by three-quarter views and confirmed the direct gaze bias for social evaluations. Further, we found a preference for highly attractive faces; increased levels of facial attractiveness might have overruled subtle gaze effects. Taken together, social in contrast to esthetic evaluations seem to be more sensitive to directions of gaze, independent of the head orientation.

#### Analysis of Faces that were Looked at by Another Face

We analyzed direct-gaze faces (0°) in social approach (DIR+LAT) and avoidance conditions (DIR+AV) to reveal whether being looked-at by another person positively increases participant’s ratings. A two-paired *t*-test (95% CI) showed a preference for direct-gaze faces that were looked-at by the second face in equal attractiveness scenes, as indicated by increased attractiveness ratings, *t*(1,62) = −3.29, *p* = 0.001. We found no preference for faces that were looked at by another face for trustworthiness ratings, *t*(1,62) = 0.72, *p* = 0.461. We did not find an effect in scenes with different levels of facial attractiveness-highly attractive faces looking toward the second, average attractive face and vice versa. These findings show that the subtle gaze effects of being looked-at, as shown by Kaisler and Leder (2016), might also be relevant in social situations with two people of average facial attractiveness. In order to focus on the interaction of head and gaze cues, we conducted Experiment 2 in which we omitted the perception of the variation of gaze direction by blurring scenes.

## Experiment 2

### Materials and Methods

#### Participants

Seventy undergraduate psychology students from the University of Vienna participated in Experiment 2 (50 female, *M*_age_ = 21.7 years, *SD* = 2.6). The experimenter explained the procedure to the participants and obtained written informed consent prior to the experiment in accordance with the ethical guidelines of the University of Vienna. The participants had normal or corrected-to-normal vision. Participants received course credits for their participation.

#### Stimuli

We used the same two-face scenes as in Experiment 1, but for the rating blocks we blurred the scenes with a Gaussian blur filter (σ = 8) in Photoshop expect for one clear ellipse (225 × 260 pixels) that showed only the face to be rated in order to direct attention toward it (**Figures 1E,F**). The gray background in the pictures matched the surrounding screen color when presented with E-Prime (V.2, Psychology Software Tools), thus effectively disappearing during stimulus presentation.

#### Design and Procedure

The design comprised a phase to familiarize participants with the scenes that varied in gaze direction of both faces. Afterward, as in Experiment 1, participants rated all 24 faces separately in two rating blocks, once for attractiveness and once for trustworthiness, 48 faces in total.

##### Familiarization phase

Participants were seated in front of a computer screen in a dimly lit room and were instructed to view scenes presented on the screen for 5000 ms and to determine the gaze direction of the left or right person. Participants responded on a standard computer keyboard with “d” for “left,” “k” for “right,” and the “space bar” for “straight ahead.” The instructions remained on screen until a response was made after which the next trial began. A single block comprised 12 distinct scenes. We presented each scene twice, once to judge the gaze direction of the left face, and once of the right face. Participants only saw each pair of unfamiliar faces in one gaze configuration. In total, 24 trials were presented in random order. Participants completed the familiarization phase and rating blocks during a single testing session lasting approximately 15 min.

##### Rating blocks

We presented the same scenes as in the familiarization phase and asked participants to rate the attractiveness and trustworthiness (blocked) of either the left or right face (randomized, counterbalanced), as described in Experiment 1. In both rating blocks, all scenes were blurred (**Figures 1E,F**). After the rating blocks, participants filled in a post-questionnaire asking about the familiarity of presented faces (data not shown). Participants who indicated that they knew any of the presented faces were excluded from the data analysis.

### Results and Discussion

#### Analysis of Gaze Conditions

Participants’ ratings were excluded for analysis when forced-direction judgments in the familiarization phase contained more than 10% errors, which accounted for elimination of 16 participants’ ratings (4.38% of all participants). From the remaining 54 participants’ judgments, we sampled across participants for attractiveness and trustworthiness judgments for each gaze direction and condition (*M* and *SD*, **Table 2**). Reliability analyses indicated internal consistency of participant’s attractiveness ratings, Cronbach’s α = 0.70, and trustworthiness ratings, Cronbach’s α = 0.89 in scenes of equal attractiveness levels. Similar for scenes with different attractiveness levels, consistency between attractiveness ratings, Cronbach’s α = 0.78, and trustworthiness ratings, Cronbach’s α = 0.86. As described in Experiment 1, we analyzed participants’ judgments of three gaze conditions (**Table 2**).

**Table 2:** Mean ratings of gaze condition in Experiment 2.

Scene	Gaze direction	Attractiveness *M* (*SD*)	ANOVA		Trustworthiness *M* (*SD*)	ANOVA	
			*p*	$\eta_{p}^{2}$		*p*	$\eta_{p}^{2}$
**Equal attractive faces**							
DIR+LAT	Direct	3.91 (1.31)	>0.05	–	**4.21 (1.37)**	0.007	0.13
	Looking at	3.79 (1.13)			3.60 (1.14)		
DIR+AV	Direct	3.95 (1.35)	0.011	0.12	4.06 (1.09)	>0.05	–
	Averted away	**4.35 (1.42)**			4.15 (1.18)		
LAT+AV	Looking at	3.99 (1.15)	>0.05	–	3.81 (1.06)	>0.05	–
	Averted away	3.88 (1.21)			4.02 (1.18)		
**Different attractive faces**							
DIR^∗^+LAT	Direct^∗^	**4.49 (1.27)**	0.001	0.29	**4.59 (1.15)**	0.001	0.22
	Looking at	3.45 (1.41)			3.65 (1.53)		
DIR^∗^+AV	Direct^∗^	**4.57 (1.12)**	0.001	0.34	**4.74 (1.30)**	0.004	0.22
	Averted away	3.60 (1.14)			3.97 (1.44)		
LAT^∗^+AV	Looking at^∗^	**4.93 (1.01)**	0.001	0.42	4.26 (1.38)	>0.05	–
	Averted away	3.86 (1.08)			4.02 (1.26)		
DIR+LAT^∗^	Direct	3.40 (1.44)	0.001	0.33	4.27 (1.25)	>0.05	–
	Looking at^∗^	**4.58 (1.22**)			4.57 (1.42)		
DIR+AV^∗^	Direct	3.18 (1.10)	0.001	0.41	3.99 (1.32)	0.074	0.07
	Averted away^∗^	**4.23 (1.21)**			4.39 (1.31)		
LAT+AV^∗^	Looking at	3.18 (1.13)	0.001	0.49	3.82 (1.27)	0.054	0.08
	Averted away^∗^	**4.53 (1.21)**			4.28 (1.56)		

*It shows mean attractiveness and trustworthiness ratings of three gaze conditions in two scene types: two average attractive faces (equal attractiveness scene), and one highly and one less attractive face (different attractive faces). Bold face indicates significant differences revealed by ANOVA. Asterisk indicates highly attractive faces in scenes of different levels if facial attractiveness.*

*^∗^Highly attractive face; bold face, statistically significant (p < 0.05); $\eta_{p}^{2}$, effect size.*

In equal attractiveness scenes, an ANOVA of attractiveness judgments (95% CI) revealed no preference for a direct or averted gaze in the social approach (DIR+LAT) and ambiguous condition (LAT+AV), but a preference for averted gaze faces looking away in the social avoidance condition (DIR+AV). Regarding social evaluations, participants considered direct-gaze faces (0°) as more trustworthy in the social approach (DIR+LAT) and showed no preference in the other conditions. In scenes of different levels of facial attractiveness, participants rated highly attractive faces as more attractive and trustworthy, independent of the direction of gaze and head orientation.

The comparison of the results of Experiment 1 with those of Experiment 2 reveals that the presence and direction of gaze plays an important role in scenes with two average attractive faces. It seems that gaze, and therefore the presence of a second person, influences effects of head angles: for example, the three-quarter views advantage vanished when gaze was blurred. Regarding trustworthiness judgments, the direct gaze bias might capture more attention than other cues, even when the gaze direction of the second person is blurred. However, in the social avoidance situation the direct gaze bias disappeared, possibly because the attention was drawn to another point outside of the scene. Regarding facial attractiveness, the ability of highly attractive faces to capture attention and elicit pleasure was not influenced by a second person or her direction of gaze.

#### Analysis of Faces that Were Looked at by Another Face

As described in Experiment 1, we analyzed direct-gaze faces (0°) in social approach (DIR+LAT) and avoidance conditions (DIR+AV) to reveal whether being looked-at by another person positively increases participants’ ratings. A two-paired *t*-test (95% CI) showed no preference for direct-gaze faces that were looked-at by the second face in equal and different attractiveness scenes, nor for highly attractive faces. As expected, subtle gaze effects of being looked-at, as shown by Kaisler and Leder (2016) may only occur when both faces and the gaze direction are clearly visible.

## General Discussion

In two experiments we studied the effects of gaze combined with different head orientations of faces-differing in attractiveness-in triadic social situations. We examined three different gaze directions that were congruent with head orientations: direct gaze in front view (0°), looking at in three-quarter view (45°), and looking away in profile view (90°). All faces were judged for attractiveness as an esthetic evaluation, and for trustworthiness as a social evaluation.

Regarding the orientation of faces, participants considered faces in three-quarter views as more attractive compared to front views (Bruce et al., 1987; Langton and Bruce, 2000), reducing the often-observed direct gaze bias (e.g., Ewing et al., 2010; Kaisler and Leder, 2016). This shows that the viewing angle influences gaze effects for esthetic evaluations of unfamiliar faces. Bruce et al. (1987) explained the advantage for three-quarter views with higher suitability for recognition compared to profile or front views. However, under very restricted presentation times, Rule et al. (2009) demonstrated judgment consistency in judgments of faces in all three orientations, and accuracy when time is limited to 50 ms (Ballew and Todorov, 2007; Rule and Ambady, 2008). Thus, we assume that the strong effects of orientation in our study were due to the unrestricted presentation time, in which the esthetic advantage of the most frequent orientation in art portraits could emerge. More importantly, by omitting gaze in Experiment 2, we showed that the effects on these kind of esthetic evaluations-i.e., of facial attractiveness-are a combination of gaze and head cues and affect observers’ judgments in triadic situations. The gaze cue, and therefore the presence of a second person, enhanced orientation effects, which is why it is important to also systematically analyze gaze effects in situations involving three (i.e., triadic situations) or more people. However, social evaluations, in our studies trustworthiness was much less affected by combined orientation effects due to the strong direct gaze bias.

Regarding faces’ attractiveness, in those cases in which a highly attractive face was paired with a less attractive face, the large difference seemed to have overwritten any gaze effect, because of the presence of a highly attractive face, which elicits high attention (Leder et al., 2016; Mitrovic et al., 2016). Neither the direction of gaze (looking at), nor the viewing angle (advantage of three-quarter views), could overcome the power of highly attractive faces in esthetic or social evaluations. Like direct gaze, facial attractiveness holds extraordinary powers and privileged attentional status. Even when attention is drawn somewhere else in the scene, facial attractiveness alters attentional deployment rapidly, effortlessly, and unconsciously (Lindell and Lindell, 2014). Among other benefits, facial attractiveness has a rewarding value. For example, we are biased to perceive direct eye contact in attractive faces (Kloth et al., 2011) and receive reward when looking at attractive faces (Aharon et al., 2001; Levy et al., 2008). This reward might indicate potential approach behavior in social interactions.

Regarding social evaluations, we found a preference for direct gaze in front views, which had previously been reported for isolated faces (e.g., Macrae et al., 2002; Mason et al., 2005; Strick et al., 2008; Ewing et al., 2010) and in triadic social scenes (Kaisler and Leder, 2016). It seems that trustworthiness builds on other social criteria than esthetic evaluations of faces, thus higher-level social judgments do not rely on structural properties of a face beyond the typicality of a face (Sofer et al., 2015). The full visibility of the eyes might be crucial for social evaluations, as we found a decrease in trustworthiness ratings in profile views which lack this availability of the eyes (McKone, 2008). Profiles of faces may be processed more as features or parts than front views, which may be processed more holistically (Rule et al., 2009). However, this may hold true for unfamiliar faces (e.g., Burton et al., 2015) raise a critical view on configural processing of familiar faces.

Finally, we found a preference for faces that were looked at by another face in scenes with two average attractive faces, as evidenced by increased attractiveness and trustworthiness judgments. This effect might result from a combined, enhanced gaze and head cue from the looking face (45°), which explains why in previous studies we did not find this esthetic preference in front views (Kaisler and Leder, 2016). This is in line with Bayliss et al. (2006), who claim that effects of preference demonstrate the flexibility of our person perception systems in guiding our social interactions and the consequences of those interactions. Our findings highlight the complex integration processes that underpin social perception of face. While attractiveness relies on distinct facial features (Main et al., 2010), social evaluations like trustworthiness relies on other impressions, such as person attributions and typicality of a face (Sofer et al., 2015). Trustworthiness judgments reflect behavioral intentions that signal approach or avoidance behaviors. Therefore, facial features such as head orientations do not affect social evaluation of others. These differences do, however, not exclude, that both types of evaluations-attractiveness and trustworthiness-elicit a certain kind of reward (Todorov et al., 2013). Todorov (2008) also argued that trustworthiness is subserved by mechanisms also underlying processing of emotional expression. However, the exact interplay with these processes requires further research, systematically including more variables.

Limitations of this study and future implications regard potential additional variables, such as effects of gender, sexual orientation, personality of the perceiver, and facial expressions. As we used only neutral expressions, future research could expand the present paradigm to include facial expressions and visual exploration of social scenes. For example, preference for isolated happy faces are stronger than faces with disgust expression (Conway et al., 2008), however we do not know how the second face in a scene might modulate observer’s evaluations in social interaction. Regarding effects of gender, previous studies revealed mixed results (see Mason et al., 2005; Conway et al., 2008). Further, in this study, we did not address participant’s sexual orientation. Sexual orientation modulates participant’s visual exploration in scenes with two faces. However, the individual facial attractiveness has the strongest influence on participant’s affective behavior in social scenes (Mitrovic et al., 2016). Therefore, in the present study, sexual orientation might be less important in scenes of highly and less attractive faces. Nevertheless, to study effects of gaze and orientation in male, female and mixed gender scenes, considering participant’s sexual orientation, would reveal a deeper understanding on people’s social evaluations and behavior. Also, additional variables such as the revised sociosexual orientation inventory (SOI; Penke and Asendorpf, 2008) have been suggested and might be considered in future studies.

## Conclusion

We showed that three-quarter views of faces enhance effects of gaze on esthetic evaluations, whereas social evaluations (i.e., trustworthiness) occur independently from head orientations. However, facial attractiveness captures attention and is privileged among other aspects in social interactions, such as head orientation and eye gaze. We therefore emphasize the differences between social and esthetic judgments; hence these evaluations rely on different facial cues and person attributions. Our study is a necessary first step to expand person perception into a more complex domain in which the multitude of factors affecting our judgments is understood.

## Ethics Statement

The study was approved by the Ethics Commitee of the University of Vienna. Decision no. 00146: there is no ethical objection to conduct the study as proposed. The experimenter explained the procedure to the participants and obtained written informed consent prior to the experiment in accordance with the ethical guidelines of the University of Vienna.

## Author Contributions

The experiments were designed and conducted by RK. The article was written by RK and HL.

## Conflict of Interest Statement

The authors declare that the research was conducted in the absence of any commercial or financial relationships that could be construed as a potential conflict of interest.
